# Effectiveness of the advisory display SmartPilot® view in the assessment of anesthetic depth in low risk gynecological surgery patients: a randomized controlled trial

**DOI:** 10.1186/s12871-022-01593-w

**Published:** 2022-02-28

**Authors:** Hilde Strand, Ann Charlott Elshaug, Øyvind Bernersen, Randi Ballangrud

**Affiliations:** 1 Department of Anesthesiology, Innlandet Hospital Trust, Sykehuset Lillehammer, Anders Sandvigs gate 17, 2609 Lillehammer, Norway; 2 Department of Anesthesiology, Østfold Hospital Trust, Sykehuset Østfold Kalnes, Kalnesveien 300, 1714 Grålum, Norway; 3 Department of Emergency, Anesthesiology and Intensive Care Unit, Innlandet Hospital Trust, Sykehuset Lillehammer, Anders Sandvigs gate 17, 2609 Lillehammer, Norway; 4 Department of Health Science Gjøvik, Faculty of Medicine and Health Sciences, Norwegian University of Science and Technology, Teknologiveien 22, 2815 Gjøvik, Norway

**Keywords:** Anesthetic depth, Advisory display, SmartPilot® View

## Abstract

**Background:**

Assessment of appropriate anesthetic depth is crucial to prevent harm to patients. Unnecessary deep anesthesia can be harmful, potentially causing acute renal failure, myocardial injury, delirium, and an increased mortality rate. Conversely, too light anesthesia combined with muscle relaxants can result in intraoperative patient awareness and lead to serious psychological trauma. This trial aimed to ascertain the effectiveness of the advisory display SmartPilot® View (SPV), as a supplemental measure in the assessment of anesthetic depth in low risk gynecological surgery patients. The hypothesis was that the use of the SPV would increase the precision of assessment, and result in a higher mean arterial pressure.

**Methods:**

This trial used a randomized, controlled, single-blind design with a homogeneous sample. Patients undergoing minor, low risk gynecological surgery were randomly assigned to two groups: a test group wherein current standards were supplemented with the advisory display SPV and a control group assessed using only the current standards. Female patients aged between 18 and 75 years with American Society of Anesthesiologists Physical Status Classification System scores of 1–3 undergoing planned general anesthesia using the total intravenous anesthetic method, combining propofol and remifentanil, were included. The exclusion criteria included a body mass index ≥ 35 kg/m^2^, a history of alcoholism, drug intake affecting propofol and remifentanil dynamics, and inability to consent. The independent sample t-test and chi-square test or Fisher’s exact test were used to assess the statistical significance of differences between the two groups.

**Results:**

A total of 114 patients were included in the analysis (test group n = 58, control group n = 56). No significant differences in the mean arterial pressure, heart rate, bispectral index, extubation delay, or post-anesthesia care unit stay were found between groups.

**Conclusions:**

The addition of the advisory display SmartPilot® View to current standards in the evaluation of anesthetic depth had no significant effect on the outcome.

**Trial registration:**

The trial was registered on January 16th 2019 with ClinicalTrials.gov (ref: NCT03807271).

**Supplementary Information:**

The online version contains supplementary material available at 10.1186/s12871-022-01593-w.

## Background

Effective anesthetic depth assessment is a crucial task involving practical clinical assessment, technology use, and experience. Traditionally, insufficient anesthetic depth has resulted in an increased focus on accidental awareness during general anesthesia. Accidental awareness can cause anxiety, depression, and post-traumatic stress disorder; however, the incidence is low [1]. Recently, excessive anesthetic depth has garnered significant attention. Intraoperative hypotension, even for a short period, may increase the risk of acute kidney failure, myocardial damage [2], and mortality [3]. Despite intraoperative hypotension being considered normal, it is suggested that the mean arterial pressure (MAP) should be maintained above 70 mmHg for autoregulation and adequate cerebral perfusion [4]. Excessive anesthetic depth may predispose patients to long-term mortality [5]. Additionally, an association between a low bispectral index value (BIS), postoperative cognitive decline, and myocardial infarction has been shown [6]. Therefore, optimization of anesthetic depth is essential to prevent these complications.

Electroencephalogram-derived devices, including the BIS™ (Medtronic, Dublin, Ireland) and GE Entropy™ (GE Healthcare, Chicago IL, USA), are commonly used for anesthetic depth assessment. However, these devices have been shown to have weaknesses [7], including the inability to monitor the area of the brain responsible for nociception. Additionally, these devices may be influenced by different artifacts, including diathermy [8]. Furthermore, the underlying physiology of the patients may affect their relationship with the clinical anesthetic depth [9]. A few bedside advisory displays, such as SmartPilot® View, have become available to support decision-making by anesthesia personnel [10]. Based on the hypnotic-opioid interaction, administered drug doses are used in response surface models to predict anesthetic depth [11]. Such advisory displays appear promising; however, few studies have validated their effect, limiting the understanding of their effectiveness. Previous studies have found that the SmartPilot® View, SPV hereinafter, (SPV; Dräger, Lübeck, Germany) and Navigator® (GE Healthcare, Chicago IL, USA) lead to reduced anesthetic drug consumption, decreased incidence of intraoperative hypotension and postoperative complications, and increased intraoperative BIS and Entropy values [12, 13]. The introduction of new technology constitutes a risk, warranting caution during use. To ascertain the benefits of these new technologies, field-based research is necessary [14].

This trial aimed to ascertain the effectiveness of the advisory display SPV, as a supplemental measure in the assessment of anesthetic depth in low risk gynecological surgery patients. The hypothesis was that the use of the SPV would increase the precision of assessment and result in a higher MAP.

## Methods

### Study design

This trial used a randomized, controlled, single-blind design with a sample of patients undergoing minor, low risk gynecological surgery. The participants were divided into two groups: a test group wherein the current standards were supplemented with the advisory display SPV, and a control group assessed using only the current standards.

### Setting and participants

**Fig. 1:**
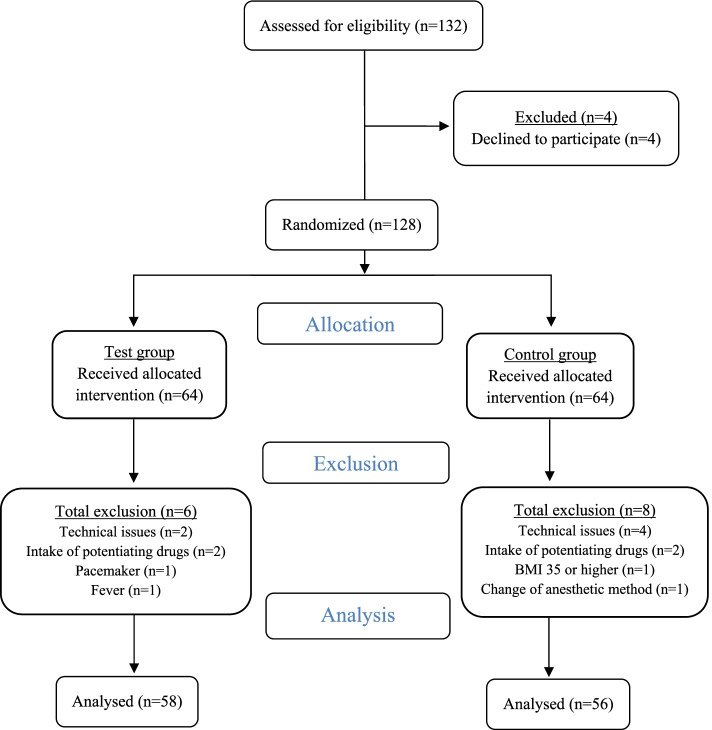
Flow diagram. Flow diagram of the enrollment process: 132 patients were assessed for eligibility, of whom four declined the invitation to participate; 128 patients were randomly assigned to the test or control groups, with 64 participants in each group. Six participants were excluded from the test group, due to technical issues, intake of potentiating drugs, pacemaker, and fever. Eight participants were excluded from the control group due to technical issues, intake of potentiating drugs, body mass index ≥35 kg/m^2^, and change in the anesthetic method. Data from 58 participants in the test group and 56 participants in the control group were included in the analysis.

The trial was performed in a surgical unit at a hospital division in a Norwegian hospital trust. The trial included in- or outpatients undergoing minor, low risk gynecological surgery. Female patients aged between 18 and 75 years with American Society of Anesthesiologists (ASA) Physical Status Classification System scores of 1–3 undergoing planned general anesthesia using the total intravenous anesthetic method, combining propofol and remifentanil, were included. The exclusion criteria included a body mass index of ≥ 35 kg/m^2^, a history of alcoholism, drug intake affecting propofol and remifentanil dynamics, and inability to consent. Initially, 132 patients were included based on the criteria; however, four patients declined the invitation to participate. The remaining 128 patients were randomly assigned to a test group (SPV) (*n* = 64) or a control group (Standard) (*n* = 64). For various reasons, 14 patients were excluded from the trial after randomization: six from the SPV group and eight from the Standard group (Fig. 1). A total of 114 patients were finally included in the trial. Figure 1 shows the enrollment process as a flow diagram.

### Randomization procedure

Block randomization was used to ensure equal distribution to both groups during the period of data collection. A total of 128 white sheets, 64 marked “SPV” and 64 marked “Standard,” were folded twice and placed in non-transparent envelopes and kept in two separate piles. Each pile of envelopes was then divided into three, resulting in six piles, with two piles of 24, two piles of 20, and two piles of 20 envelopes. The two piles of 24 were mixed together in a large, sealed envelope by two colleagues. The same procedure was followed with the piles of 20. Finally, the large envelopes containing 48 (first block) and two sets of 40 (second and third blocks) envelopes were obtained and marked with the number of envelopes they contained. Each block was finished before opening another block. At all times, envelopes were stored in locked cabinets available only to selected individuals.

Cohen’s d was used as the effect size because there were no previous studies from which the standard deviation for the primary target parameter could be determined [15]. The sample size calculation for the independent t-test of the primary outcome parameter was done with the program G*Power. For a medium effect (Cohen’s d = 0.5) and power of 80% at a significance level of 0.05, the sample size per group was 64 and thus a total of 128 subjects [16].

### Intervention

During the data collection period, an agreement was established with the nursing staff of the gynecology ward to brief and obtain the written consent of all eligible patients scheduled for surgery. However, some patients had not been introduced to the trial on the day of surgery for various reasons. In such instances, patients was given an orientation and an invitation to participate in the trial. This occurred more often with outpatients because their consultations were on the same day as the procedure. Following hospital procedure, routine preoperative medication was administered to the patients. After ensuring eligibility and consent by the patient, an envelope was drawn from the current block to allocate the patient to a group. The investigators were aware of the method in use, naturally; however, the patients were blinded to group allocation and unable to influence the data due to unconsciousness.

The Schnider and Minto models were applied for propofol and remifentanil, respectively, with target-controlled infusion in effect site mode using Alaris PK® syringe pumps (BD Medical, Franklin Lakes, NJ, USA) [10]. A standard for induction was set to ensure a standardized starting point using an effect site concentration of 6 µg/mL for propofol and 4 ng/mL for remifentanil. During induction, the dosage appropriateness was evaluated consecutively and adjusted accordingly. Before intubation, 0.6 mg/kg rocuronium bromide or 0.1 mg/kg vecuronium bromide was administered as a single bolus to ensure the best possible intubation conditions. The standard method of measurement used in both groups was in accordance with the Norwegian standard of anesthesia [17]. The guideline states that patients under general anesthesia should, as a minimum, be monitored using electrocardiogram, capnography, pulse oximetry, and blood pressure. In addition, temperature, degree of muscle relaxation, and BIS were monitored. The core temperature was measured in the esophagus using Level 1® Temperature probes (Smiths Medical, Minneapolis, MN, USA). The degree of muscle relaxation was measured using the train-of-four (TOF) test, using TOFscan® (Dräger, Lübeck, Germany). The BIS^™^ Quatro Sensor (Medtronic, Dublin, Ireland) was used to measure BIS, with a target range between 40 and 60, as per the manufacturer’s instructions [18].

### Guidance of the advisory display SPV

The advisory display SPV version 3.11.8.0 (SPV; Dräger, Lübeck, Germany) was used in the test group. The SPV estimates anesthetic depth based on the effect site concentration through the connected syringe pumps, gas concentration values from the ventilator and also manually entered boluses of neuromuscular blocking agents, fentanyl, midazolam, sufentanil, and alfentanil [10]. This version did not integrate values coming from the patient, such as heart rate (HR), MAP, BIS etc. The presented anesthetic depth is an estimate based on the given drugs, and not on measurements from the patient. The SPV was used according to the manufacturer’s instructions [10]. The syringe pumps were connected to the anesthesia ventilator to calculate the synergistic effect of propofol and remifentanil. The calculation was graphically displayed in a two-dimensional diagram, as guidance to understand the balance between the administered drugs and adjust the anesthetic depth accordingly. The drug dosage data are turned into visual information, placing the anesthetic depth in isobole lines to indicate the present level, including a prediction for the next 20 min [10].

The SPV was actively used from the commencement of surgery until the patient awakened, but not as a tool for predicting tolerance of laryngoscopy or placement of laryngeal mask. During surgery, the anesthetic depth of the patient was kept within the dark grey isobole, named “tolerance of laryngoscopy (TOL) 50 and 90,” i.e., predicting the TOL in 50–90% of patients. This is, according to the manufacturer’s instructions, the desired area for patients to be in during surgery, as this area predicts tolerance of surgical stimuli [10]. The SPV includes a noxious stimulation response index (NSRI), which was given attention, predicting the probability of patient response to certain stimuli, on a scale ranging from 0 to 100.

According to the instructions, the SPV user must always administer drugs appropriate to the clinical assessment. For participant safety, an agreement was made to ignore the SPV if the advice provided seemed clinically inappropriate, despite placement in the desired area.

BIS was already included in the current standard and was kept as a measurement and taken into consideration in both groups. For patient safety it was difficult to blind the investigators to parameters that were already in use in the current standard. Numerical BIS values, as the only available option, were used to compare the groups. If the blood pressure and/or HR demonstrated considerable decline, the situation was assessed to determine appropriate bolus administration of either atropine 0.5 mg, ephedrine 5 mg, or phenylephrine 100 µg. If BIS was low at the same time, drug dosage was also adjusted. In the test group, the adjustments were made to keep the patient in the desired area suggested by the SPV. At the end of the surgery, the doses of propofol and remifentanil were reduced, if possible. Waking of patients was attempted once every minute through careful shaking and loud enunciation of their names. Fentanyl was administered immediately after extubation or removal of the laryngeal mask to avoid affecting the extubation delay.

### Data collection

Data was collected between January and November 2019. Two operating rooms, with identical medical equipment, were utilized for these surgeries. H.S. and A.C.E. were the only investigators assessing the participants’ anesthetic depth and collecting data. The primary outcome was MAP (mmHg). Secondary outcomes were HR (beats/min), BIS (value 0–100), total drug dose (mg/kg/min propofol, µg/kg/min remifentanil, mg atropine and ephedrine, and µg phenylephrine), extubation delay (min), and post-anesthesia care unit (PACU) time (min). MAP, HR, and BIS were measured every 3 min. At the end of the surgery, the total drug dose was recorded manually from the patient’s case file. The period between the syringe pump shutdown and patient eye-opening was considered “extubation delay,” which was measured in minutes. The period between arrival and departure from the PACU was considered “PACU time.”

### Statistical analysis

The data were analyzed using SPSS Version 26 (IBM Corp., Armonk, NY, USA). The variables were normally distributed and analyzed descriptively using frequencies, percentages, means, and standard deviations. The independent sample t-test was used to compare group means of the patients’ demographic characteristics and duration of general anesthesia and surgery. It was also used to compare the group means of the primary and secondary outcomes. The means of MAP, HR, and BIS were calculated with the values occurring between skin incision and skin closure. Cohen’s d determined the effect size of the mean difference, where 0.2 corresponds to a small effect; 0.5, medium effect; and 0.8, large effect [19]. The chi-square test for independence was used to explore the number of ephedrine, atropine, and phenylephrine administrations, with the phi coefficient being used to determine the effect size. Fisher’s exact probability test was used to analyze the distribution of ASA scores and surgical procedure between the groups due to violations in the assumption of a frequency of ≥ 5 in at least 80% of the cells. In this instance, Cramer’s V was used to determine the effect size. In all tests, statistical significance was set at *P* < 0.05. The trial was reported according to the CONSORT 2010 checklist (Additional file).

## Results

### Patient characteristics

The demographic characteristics of the patients are shown in Table 1. No significant difference was found between the two groups.

**Table 1:** Participant characteristics: comparison between test and control groups

Variable	Measure	Test group (*n* = 58)	Control group (*n* = 56)	Mean difference	CI		*p*	ES
					Lower	Upper		
Age (years)	mean (SD)	48.52 (14.81)	50.45 (12.42)	1.93	−3.15	7.01	0.45^a^	0.14^b^
Weight (kg)	mean (SD)	70.60 (11.03)	72.21 (12.22)	1.61	−2.71	5.93	0.46^a^	0.14^b^
Height (cm)	mean (SD)	167.95 (5.38)	167.43 (5.26)	−0.52	−2.50	1.46	0.60^a^	0.10^b^
Body mass index (kg/m^2^)	mean (SD)	24.95 (3.44)	25.68 (4.03)				0.30^a^	0.20^b^
Baseline MAP (mmHg)	mean (SD)	97.78 (17.30)	97.64 (15.04)	−0.133	−6.16	5.90	0.97	0.16 ^b^
Surgery duration (min)	mean (SD)	72.53 (43.28)	67.02 (47.57)	−5.51	−22.38	11.35	0.52^a^	0.12^b^
Anesthesia duration (min)	mean (SD)	100.72 (46.34)	93.82 (52.20)	−6.90	−25.20	11.40	0.46^a^	0.14^b^
ASA score							0.59^c^	0.14^d^
1	n (% of total)	30 (51.9)	28 (48.3)					
2	n (% of total)	26 (48.1)	28 (51.9)					
3	n (% of total)	2 (100)	0 (0)					
Surgery type							0.66^c^	0.12^d^
Laparoscopy	n (% of total)	39 (54.2)	33 (45.8)					
Hysteroscopy	n (% of total)	9 (40.9)	13 (59.1)					
Vaginal plastic surgery	n (% of total)	9 (52.9)	8 (47.1)					
Unspecified/Other	n (% of total)	1 (33.3)	2 (66.7)					

Patient characteristics including age, weight, height, body mass index, surgery duration, anesthetic duration, ASA score, and type of surgery compared between the test and control groups. There were no significant differences between the two groups for any characteristics

*ASA *American Society of Anesthesiologists, *CI *confidence interval, *ES *effect size

^a^ Independent sample t-test

^b^ Cohen’s d

^c^ Fisher’s exact test

^d^ Cramer’s V

### Primary outcome

The mean MAP in both groups was 75 mmHg with a difference of 0.81; however, the difference was not significant. Additionally, the number of minutes where MAP was < 60 mmHg was not significant between the groups; the control group had a mean time of 4.45 min, and the test group was 5.2 min (Table 2).

**Table 2:** Primary and secondary outcome variables: comparison between test and control groups

Variable	Measure	Test group (*n* = 58)	Control group (*n* = 56)	Mean difference	CI		*p*	ES
					Lower	Upper		
MAP (mmHg)	mean (SD)	75.64 (8.90)	74.83 (10.85)	−0.81	−4.49	2.87	0.97^a^	0.01^b^
MAP < 60 mmHg duration (min)	mean (SD)	5.22 (10.77)	4.45 (9.06)	−0.78	−4.48	2.91	0.68^a^	0.08^b^
BIS value	mean (SD)	47.52 (6.22)	45.95 (6.80)	−1.57	−3.99	0.85	0.39^a^	0.16^b^
BIS < 40 duration (min)	mean (SD)	12.81 (18.03)	14.89 (21.11)	2.08	−5.19	9.36	0.57^a^	0.11^b^
HR (beats/min)	mean (SD)	59.63 (7.50)	58.11 (8.51)	−1.52	−4.49	1.45	0.60^a^	0.10^b^
Extubation delay (min)	mean (SD)	5.69 (2.15)	5.96 (2.79)	0.28	−0.65	1,20	0.56^a^	0.11^b^
Time in PACU (min)	mean (SD)	140.22 (84.10)	137.45 (73.94)	−2.79	−32.21	26,65	0.86^a^	0.04^b^
Propofol (mg/kg/min)	mean (SD)	0.10 (0.02)	0.12 (0.11)	0.02	−0.01	0.05	0.17^a^	0.25^b^
Remifentanil (µg/kg/min)	mean (SD)	0.17 (0.05)	0.18 (0.06)	0.01	−0.01	0.03	0.28^a^	0.21^b^
Ephedrine (mg)	mean (SD)	2.28 (6.04)	2.68 (4.42)	0.39	−1.58	2.36	0.69^a^	0.07^b^
Atropine (mg)	mean (SD)	0.06 (0.23)	0.07 (0.22)	0.01	−0.73	0.10	0.80^a^	0.05^b^
Phenylephrine (µg)	mean (SD)	3.45 (26.26)	3.57 (26.73)	0.12	−9.71	9.96	0.90^a^	0.00^b^
Participants receiving ephedrine, atropine, or phenylephrine	n (% in group)	16 (27.6)	21 (37.5)				0.26^c^	−0.11^d^

Primary and secondary outcome variables and comparison of the outcomes between the test and control groups, including mean MAP, mean duration of MAP < 60 mmHg, mean BIS value, mean duration of BIS value < 40, mean HR, mean duration of extubation delay, mean duration of PACU stay, mean dosage of propofol, remifentanil, ephedrine, atropine, and phenylephrine. The last row shows the number of participants in each group that needed a bolus dose of ephedrine, atropine, or phenylephrine. Fewer participants in the test group received bolus doses compared to the control group (n = 16 and n = 21, respectively), but no significant differences were found between the groups in any of the outcome measures assessed

*BIS* bispectral index, *CI *confidence interval, *ES *effect size, *HR *heart rate, *MAP *mean arterial pressure, *PACU *post-anesthesia care unit

^a^ Independent sample t-test

^b^ Cohen’s d

^c^ Pearson’s chi-square test

^d^ Phi coefficient

### Secondary outcomes

Statistically significant differences were not found between the groups’ mean HR or BIS. The results show that the use of propofol and remifentanil was less in the test group, corresponding to a small effect, but the difference between the groups was not significant. Additionally, fewer patients in the test group received bolus administration of atropine, ephedrine, and phenylephrine, but the difference was not significant. No significant difference was found in the extubation delay and PACU times between the two groups. Furthermore, the standard deviation of PACU time was large, but this applied to both groups. No harm or unintended effects were found in any of the patients (Table 2).

## Discussion

This trial aimed to ascertain the effectiveness of the advisory display SPV as a supplemental measure in determining anesthetic depth, by comparing the use of the SPV with the current standard in low risk gynecological surgery patients, hypothesizing a higher MAP as the primary outcome. No significant differences were found between the groups in terms of the primary or secondary outcomes.

There are a limited number of trials on the use of the advisory display SPV, all with different primary outcomes including recovery-time, consumption of volatiles, and time in the optimal anesthetic zone. Compared to the mentioned trials, the current trial was the only one using total intravenous anesthesia (TIVA), while the others used a combination of volatiles and intravenous anesthesia [12, 13, 20]. The patient population in Cirillo et al. [12] and Marimoto et al. [20] consisted of ASA 1–2 patients, like most patients in the current trial. In the LeBlanc et al. [13] trial, almost half of the study population consisted of ASA 3 patients. The starting point is not well described in the trials, making comparison difficult. Protocol compliance is not mentioned. In LeBlanc et al. [13], the investigators were blinded to the BIS, whereas Marimoto et al. [20] included BIS in the intervention and describes some of the issues mentioned in the current trial.

Although knowledge of drug disposition and effect are essential in the practice of anesthesia, it remains difficult to extrapolate such principles into clinical practice [19]. The assessment of anesthetic depth may have been incorrect in the past (before the trial), leading to an increase in the depth of anesthesia in these patients. The current trial did not demonstrate a reduction in drug consumption, unlike previous studies [12, 13, 20]. The results suggest that the benefit of using SPV may be limited to certain groups of patients. Until now, only LeBlanc et al. [13] have been able to establish an association between SPV use and more beneficial hemodynamics. Their trial included older and more fragile participants compared to the current trial. They suggested that the use of the SPV may be more relevant to the elderly, who are already prone to hypotension.

Additionally, the benefit of use of the SPV might be limited to certain anesthetic methods. Cirillo et al. [12], LeBlanc et al. [13] and Marimoto et al. [20] all used a combination of inhaled and intravenous anesthetics. The supplemental application of SPV may be effective when a combination of several drugs or continuous pressors is used. Furthermore, the starting point is essential in judging the effectiveness of SPV. Yet, the degree of change to induce statistical significance remains uncertain. The estimated calculation from the SPV seemed to fit most often, demonstrating that the starting point was already within the recommended area, without a need for change. This may indicate that the effectiveness of SPV was limited only in the context of the current trial. As mentioned, the starting point in the previous trials [12, 13, 20] are not well described, making comparison difficult.

In addition, using BIS may have affected the effectiveness of SPV. Being used to assessing BIS as an indicator of the patient’s anesthetic depth may have influenced the interpretation. This issue was also mentioned by Marimoto et al. [20]. Without BIS, users will probably depend more on the SPV. Nevertheless, the trial was considered closer to reality if BIS was kept as current standard. Despite its weaknesses, BIS provides a direct measurement of the patient status, unlike SPV, which provides an estimate. Measuring BIS in both groups was considered more realistic, and was necessary, considering that the SPV was a supplementary device [10] and did not replace BIS. Still, it must not be forgotten that BIS only measures the hypnotic component of anesthetic depth, whereas the NSRI in SPV promises to predict the analgesic component [19].

From the perspective of learning, the effectiveness of advisory displays, including SPV, may be highlighted. Two simulation studies showed that the use of such advisory displays helped achieve better hemodynamic control and faster waking time in patients [20]. In addition, the anesthesiologists’ mental demand, effort, and frustration levels were reduced, indicating that such advisory displays are a viable supplemental method to anesthesia monitoring [21].

Regardless, models for the predicted effect are based on samples, not the whole population [7, 8]. Additionally, the sample tends to consist of healthy, male volunteers with a mean age of approximately 50 years [10]. Future studies should involve a larger sample and investigate the application of such advisory displays with other patient groups and anesthetic methods. Furthermore, the subjective experience and opinions of anesthesia personnel regarding the use of such advisory displays should be explored. Finally, future studies should have a more comprehensive approach, including some form of volume status assessment.

To improve the SPV software, data should be based on bigger samples with a larger age range. This would make the estimation more precise. In addition, changes in surgical stimuli should be considered, if possible.

### Strengths and limitations

The homogeneity and comparability of the sample strengthened the internal validity of the trial, making the results more credible and trustworthy. Despite that, the current trial has several limitations. First, protocol compliance was not measured. The desired area between TOL 50 and 90 seemed “wide,” allowing decent changes of effect site concentrations, hence giving no reason to ignore SPV in terms of turning it off. Still, there was no quantification of how many times TOL was out of range. Second, not achieving the estimated sample size affects the power, to some extent. Third, the concomitant use of SPV and the current standard over a period may have unintentionally increased the skills in performing these procedures, which may have affected the reliability of the trial. To overcome this, the trial could have been designed differently. Still, a randomized controlled trial design is less prone to bias and provides a greater strength of evidence than non-randomized study designs. In trials such as this one, there will always be a degree of discretion, due to participants needing individualized care. Following a stringent protocol is desirable, but not always possible. Fourth, double blinding was not possible since the method in use was visible to all healthcare workers. Only the patients were blinded because influencing data was not possible when unconscious. Finally, involving only female participants may have negatively affected the external validity and generalizability of the trial [19] as the results are only applicable to this population.

## Conclusions

Assessing the anesthetic depth in low risk gynecological surgery patients, using SPV as a supplement to the current standard, had no significant effect on primary or secondary outcomes. The lack of difference may be due to low protocol compliance. Its effectiveness remains uncertain and may be affected by several factors, including the patient group, anesthetic method, and starting point.

## Supplementary Information

**Additional file 1.**

## Data Availability

The datasets used and/or analyzed during the current study are available from the corresponding author on reasonable request.

## Abbreviations

ASA: American Society of Anesthesiologists Physical Status Classification System
BIS: Bispectral index
HR: Heart rate
MAP: Mean arterial pressure
PACU: Post-anesthesia care unit
SPV: SmartPilot® View
TOF: Train-of-four
TOL: Tolerance of laryngoscopy
